# Prospects of Neutralizing Nanobodies Against SARS-CoV-2

**DOI:** 10.3389/fimmu.2021.690742

**Published:** 2021-05-28

**Authors:** Fangfang Chen, Zhihong Liu, Fan Jiang

**Affiliations:** ^1^ Department of Pharmacy, Shenzhen Hospital, Southern Medical University, Shenzhen, China; ^2^ State Key Laboratory of Chemical Oncogenomics, School of Chemical Biology and Biotechnology, Peking University Shenzhen Graduate School, Shenzhen, China; ^3^ NanoAI Biotech Co., Ltd., Huahan Technology Industrial Park, Shenzhen, China

**Keywords:** nanobodies (VHH), SARS-CoV-2, phage display, receptor binding domain, neutralizing

## Abstract

Since December 2019, the SARS-CoV-2 has erupted on a large scale worldwide and spread rapidly. Passive immunization of antibody-related molecules provides opportunities for prevention and treatment of high-risk patients and children. Nanobodies (Nbs) have many strong physical and chemical properties. They can be atomized, administered by inhalation, and can be directly applied to the infected site, with fast onset, high local drug concentration/high bioavailability, and high patient compliance (no needles). It has very attractive potential in the treatment of respiratory viruses. Rapid and low-cost development of Nbs targeting SARS-CoV-2 can quickly be achieved. Nbs against SARS-CoV-2 mutant strains also can be utilized quickly to prevent the virus from escaping. It provides important technical supports for the treatment of the SARS-CoV-2 and has the potential to become an essential medicine in the toolbox against the SARS-CoV-2.

## Introduction

Since December 2019, a novel, highly transmissible severe acute respiratory syndrome coronavirus 2 (SARS-CoV-2, COVID-19) (1, 2) has erupted on a global scale. As of February 2021, more than 100 million people have been infected and more than 2.5 million lives have been claimed. These numbers are still rising, and there are still nearly 400,000 new confirmed cases every day. The global COVID-19 pandemic poses serious challenges to patients, health care systems, and economic and social activities. Although isolation and preventive measures can help curb the spread of the virus, it is easy to rebound after social restrictions are lifted. Countries around the world are gradually advancing the use of the SARS-CoV-2 vaccine, but the vaccine may not be suitable for patients with weak immunity system. It is still essential need to provide additional methods for the prevention or treatment of high-risk patients and children. Therefore, neutralizing antibodies or related molecules have great potential as direct antiviral drugs (3).

Early treatment of SARS-CoV-2 with convalescent plasma (CP) can effectively prevent progressive clinical deterioration (4). However, the survivors have limited plasma supply with a risk of infection and allergies. Potent neutralizing monoclonal antibodies (mAb) isolated from patients with COVID-19 that can be recombinantly produced has been developed for passive immunotherapy (5–10).

Although monoclonal antibody-based therapy helps patients with mild symptoms of COVID-19, it still requires extremely high doses, usually a few grams intravenously (11, 12). The need for high-dose monoclonal antibodies for effective neutralization may reflect the virulence, pathogenesis of COVID-19 and the low efficiency of intravenous administration. When treating lung infections, these relatively large biomolecules pass the plasma-lung barrier with low efficiency (13). In addition, traditional monoclonal antibodies cannot be produced quickly and at low cost, and antibody drugs cannot be rapidly developed against mutant virus strains, and they are not easy to optimize. They cannot target multiple specific epitopes. Antibody-dependent enhancement (ADE) must be evaluated, for the possibility of infection (14). Antibody-dependent enhancement of infection means that low-quality non-neutralizing antibodies bind to virus particles through its Fab domain, and the Fc domain binds to the Fc receptor (FcR) of monocytes or macrophages to promote virus entry and infection. Meanwhile, the high costs and challenges associated with the mass production of monoclonal antibodies may limit the clinical applications of monoclonal antibodies (15).

**Figure 1:**
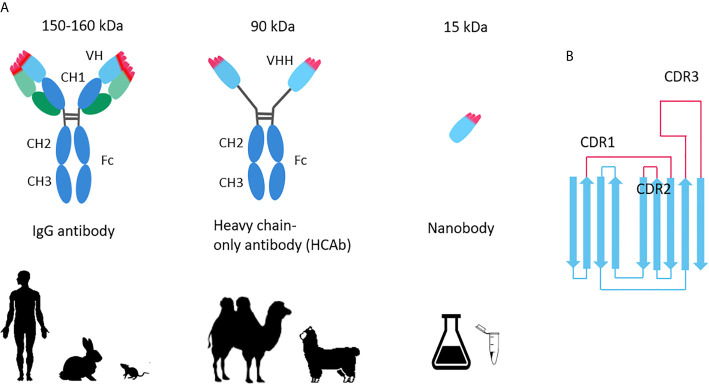
**(A)** Comparison of conventional antibody, heavy chain antibody and Nanobody; **(B)** Nanobody topology.

In contrast, the variable domains of heavy-chain–only antibodies (VHHs) derived from camelid animals—called Nanobodies (**Figure 1**), or single domains antibody (sdAb), with a molecular weight of only 12–15 kDa, is only one-tenth of the conventional monoclonal antibody (about 150–160 kDa), but it can specifically bind to various antigens like traditional antibodies. Nanobodies provide possible opportunities for rapid production of antiviral drugs.

## Research Prospects of SARS-CoV-2 Nanobodies

Although the general structural features of Nanobodies (Nbs) are similar to that of human variable heavy domain (VH), in fact, four major amino acid substitutions are observed in framework region 2 (FR2), and more hydrophilic amino acids (16) are used instead of hydrophobic residue (involved in the VH/VL interaction in IgG antibodies). These substitutions gave Nbs higher solubility. In addition, they have a long CDR3 loop, which can increase the size of the antigen-binding loop and can bind to concave epitopes that traditional antibodies cannot recognize (17, 18). The longer CDR3 and extended CDR1 are compensating for absence of the three CDRs of the VL, compensated for the absence of the three CDRs of the variable light domains (VL). These special structure features increase their stability and solubility, even under harsh conditions or high temperatures (19–22).

Nbs have several important advantages over traditional antibodies, including their excellent biochemical properties: high thermal stability, high solubility, and easier penetration into tissues (19). They can be expressed in prokaryotic systems with lower production costs (23, 24). Although for therapeutic applications, it will be necessary to produce under GMP, which may increase the production cost to a level comparable to mAb production. Nbs can be easily bioengineered into novel bivalent/multivalent/multispecific and high-affinity molecules (25, 26). These advantages make them a powerful tool for immune-therapy, immune-diagnosis, and immune-analysis (25, 27, 28).

The first Nanobody drug, Cablivi, was approved by the EMA/FDA in 2018/2019 for the treatment of acquired thrombotic thrombocytopenic purpura (aTTP) adult patients. Nbs are currently widely evaluate in preclinical or clinical use in various diseases, such as brain tumors, inflammation, and lung diseases (28–32), some have entered various phases of clinical development. The prospect that Nbs can be fused with Fc domains, peptide tags or other Nbs, as well as with nanomaterials, radionuclides, photosensitizers, etc., has broadened its application range (25, 27).

The development of therapeutic agents against various viral infections is an interesting and promising research field. For example, Nbs against dengue fever virus (33), hepatitis B virus (34), hepatitis C virus (35), polio virus (36), norovirus (37), Ebola virus (38), anti-HIV anti-CXCR4 monovalent and bivalent (39) and anti-p24 monovalent and bivalent (40) Nbs, anti-rabies virus bivalent/albumin-linked Nbs (41) and anti-VP6 as a possible effective prevention method for rotavirus A-related diarrhea (42) has been documented.

Nbs have excellent physical and chemical properties. They can be atomized, administered by inhalation, and directly applied to the infected site with high local drug concentration/high bioavailability, and high patient compliance (no needles). It has very attractive potential in the treatment of respiratory viruses (43, 44). In recent years, research on their application against respiratory pathogens has also accelerated. For example, the use of Nbs against MERS-CoV (45), H1N1 (46), H5N1 (47), influenza (48) and so on. ALX-0171, a trivalent Nanobody (Nb) that neutralizes Respiratory syncytial virus (RSV), directly prevents or treats RSV infection in the lungs of cotton rats, and effectively reduces the RSV titer in the nose and lungs of cotton rats (44). The ALX-0171 clinical trial found that despite the lack of significant improvement in the clinical outcome of treatment a few days after the onset of symptoms (49), high-quality Nbs are still promising drug candidates for the treatment of COVID-19 pneumonia (50–52). The development of highly effective anti-SARS-CoV-2 Nbs may provide an important means for multifunctional, cost-effective prevention, treatment and immediate diagnosis.

Like other zoonotic coronaviruses (SARS-CoV-1, MERS), the new coronavirus expresses a surface Spike (S) glycoprotein, which is composed of two subunits, S1 and S2, and forms the homotrimeric S protein (53). This homotrimeric S protein can interact with host cells. The interaction between the new coronavirus and the host cell is mediated by the receptor binding domain (RBD) of the S1 subunit, which binds to the peptidase domain (PD) of Angiotensin-converting enzyme 2 (ACE 2) (53). After that, the S2 subunit undergoes a drastic conformational change and triggers membrane fusion (53). The binding affinity of the new coronavirus S protein to ACE2 (Kd is 15 nM) is 10–20 times higher than that of the SARS-CoV-1 S protein, which is speculated to be the reason why the SARS-CoV-2 is more infectious (54, 55).

Structural studies have shown that the conformational flexibility of the SARS-CoV-2 trimer S protein allows each of its RBD to exist in two main conformations: the “down” conformation is not easy to be accessed by ACE2 or most neutralizing antibodies (NAb), and the “up” conformation is easily bound by ACE2 and most NAb (54, 56, 57). Many Nbs compete with ACE2 to bind to the S protein of RBD in an upward conformation, thereby preventing viral infection (10, 58). Some NAbs can bind to and stabilize the “down” conformation of the S protein, thereby preventing the conformational changes required for the virus to enter the host (8, 59).

At present, higher affinity SARS-CoV-2 neutralizing Nbs are mainly obtained through the “*in vivo*” method (**Table 1**). The recombinant S protein or RBD protein is used to immunize experimental animals such as camels or alpacas. The antibodies produced by B cells undergo affinity maturation *in vivo*. Isolated B cells and constructed immune library. Phage display, yeast display, ribosome display and other technologies are used to screen SARS-CoV-2 Nbs. Several high-affinity neutralizing SARS-CoV-2 Nbs have been identified through these *in vivo* methods (60–65), and the half-inhibitory concentration IC50 of some Nbs that neutralize SARS-CoV-2 lower than pmol (62).

**Table 1:** SARS-CoV-2 Neutralizing Nanobodies (Nbs).

References	Nanobody name	Co-crystal structure with RBD	Cryo-EM structure with Spike protein	Neutralizing pseudovirus (IC50)	Affinity for RBD (Kd, tested by SPR)	Method (Source)	Framework region (FR)
(60–61)	VHH-E	7KN5		60 nM	1.86 nM	Immune library + phage display	unhumanized
	Tri-VHH-E			0.17 nM			
(62)	Nbs 89			0.133 nM	108 pM	Immune library + MS proteomic strategy	unhumanized
	Nbs 20		7JVB	0.102 nM	10.4 pM		
	Nbs 21		6VXX	0.045 nM	<1 pM		
	Tri-Nb 20			4.1 pM	<1 pM		
	Tri-Nb 21			1.3 pM	<1 pM		
(63)	Ty1		6ZXN	54 nM	5-10 nM	Immune library + phage display	unhumanized
	Ty1-Fc			1 nM			
(64)	Nb11-59			36.7 nM	21 nM	Immune library + phage display	unhumanized
(65)	VHH-72	6WAQ			38.6 nM	Immune library + phage display	unhumanized
	Bi-VHH 72			13.3 uM			
(69)	H11-D4	6YZ5	6Z43		39 nM	Naïve library	unhumanized
	H11-H4	6YBP	6ZHD		12 nM		
	H11-D4-Fc			22 nM			
	H11-H4-Fc			6 nM			
(59)	Nb6		7KKK	2.0 uM	210 nM	Synthetic library + yeast display	unhumanized
	mNb6	7KKJ		6.3 nM	0.45 nM		
	Tri-mNb6		7KKL	0.12 nM	<1 pM		
(73)	Sb23			40nM		Synthetic libraries (concave, loop and convex) + phage display	unhumanized
(74)	n3021				0.63 nM	Synthetic library + yeast display	humanized
(75)	Sb15				24.22 nM	Synthetic libraries (concave, loop and convex) + phage display	unhumanized
	Sb68				37 nM		

At the earliest, scientists from the University of Texas, the National Institutes of Health, and Ghent University used SARS-CoV-1 S (SARS) and MERS-CoV S proteins to subcutaneously immunize llamas, using SARS-CoV-1 S or MERS-CoV S protein was used in two rounds of panning by phage display, and obtained several Nbs against S protein (65). These Nbs can neutralize MERS-CoV or SARS-CoV-1 S pseudovirus, respectively. They also found that VHH72 had cross-reactivity, targets both SARS-CoV-1 S and SARS-CoV-2 S protein. They engineered VHH72 into a bivalent human IgG Fc fusion and proved that the bivalent VHH72 can neutralize SARS-CoV-2 S pseudovirus more efficiently (65). This study proved the rationality of the large-scale production of Nb–Fc fusions in a commercial standard CHO cell system. VHH-72-Fc exhibits ideal biophysical properties and effective neutralization potential (65), and may be a potential therapeutic candidate drug. This research field is attracting more and more attention.

Wan Yakun, from Shanghai Luoqi Biomedical Technology Co., Ltd., Shanghai, and cooperators then immediately reported their findings. They immunized four camels with SARS-CoV-2 recombinant RBD, after that, three rounds of phage display biopanning, periplasmic extraction ELISA was performed (PE-ELISA), they finally identified 381 SARS-CoV-2 RBD binding Nbs, of which Nb11-59 showed strong antiviral activity against real SARS-CoV-2, and the ND50 was 0.55 μg/ml (64). Nb11-59 can be produced on a large scale in Pichia pastoris and shows good stability. It is expected to be developed into an inhalable drug to treat COVID-19 (64).

Hanke et al. immunized alpaca with SARS-CoV-2 S1-Fc and RBD, and performed two consecutive rounds of phage display. Nanobody Ty1 was isolated based on ELISA binding and screening. Ty1 can specifically bind to the RBD of SARS-CoV-2 spike glycoprotein (63). Ty1 binds to RBD with high affinity, and blocks the binding of RBD to ACE2. The structure of cryo-electron microscopy revealed that Ty1 binds to RBD either in “up” or “down” conformations, and spatially hinders the binding of RBD-ACE2 (63). Ty1 can be expressed in large quantities in bacteria (63), providing opportunities for large-scale production, and is an excellent drug candidate for anti-COVID-19.

Researchers are optimistic about the synergistic application of different SARS-CoV-2 neutralizing antibodies (especially neutralizing antibodies that compete with ACE2). Researchers further try to design multivalent and multi-specific Nbs to neutralize SARS-CoV-2. Koenig et al. used the RBD of the SARS-CoV-2 and the formalin-inactivated SARS-CoV-2 to immunize the alpaca. Through phage display, four Nbs (VHHs E, U, V and W) were found to effectively neutralized SARS-CoV-2 and SARS-CoV-2 pseudotyped viruses (60). These four Nbs bind to two different epitopes on RBD. “UVW” targets the same epitope, and “E” targets another epitope. These two groups of Nbs can inhibit infection through a synergistic effect (60). They designed a variety of bivalent, trivalent, and multi-specific Nbs to improve the neutralization efficiency. The experiments found that VHH EEE (trivalent) most effectively inhibited the infection (IC50 of neutralizing SARS-CoV-2 is lower than pmol) without activation virus fusion and may inactivate the virion by inhibiting the interaction of the virus with its receptor (60). VHH VE targets two independent epitopes at the same time, which prevents the emergence of resistance escape mutants in evolutionary experiments (60).

Yufei Xiang, from the University of Pittsburgh and cooperators immunized camels with recombinant SARS-CoV-2 RBD, used proteomics methods and mass spectrometry and identified a large number of high-efficiency SARS-CoV-2 neutralizing Nbs (62). It was found that several Nbs neutralized SARS-CoV-2 at very low doses, the IC50 is lower than pmol (62). They constructed multivalent Nb and achieve ultra-high neutralization efficiency (IC50 as low as 0.058 ng/ml) and may prevent mutations virus strains from escaping (62). These heat-stable Nbs can be quickly mass-produced by microorganisms, and are resistant to freezing, drying and aerosolization (62). They further developed the most efficient tri-valent Tri-Nb21 into PiN-21 aerosol, which can effectively prevent and treat Syrian hamsters infected by SARS-CoV-2 at an ultra-low dose, greatly reduce the viral load, prevent lung damage and viral pneumonia (61, 62).

Viruses are prone to mutation. Several RBD mutations observed in the Global Avian Influenza Data Sharing Initiative (GISAID) (66), and the mutation rate may increase under the large-scale epidemics and drug treatments. If the target sites of existing vaccines or drugs are mutated, these vaccines or drugs may fail. At present, several SARS-CoV-2 variants are spreading globally, including the N501Y mutant with RBD mutation (67). However, the “*in vivo*” screening method need a long development period (usually >3 months) from antigen to the final specific Nbs. So, it is difficult to develop and generate antibodies against new virus mutant strains or new viruses quickly and at low cost. Therefore, rapid and efficient “*in vitro*” screening has become an important technical means.

“*In vitro*” antibody screening mainly relies on large antibody libraries (naïve libraries or synthetic libraries), which can target almost all antigen proteins, faster and at low cost. The source of naïve library is the B lymphocytes of camelid animals that have not been immunized with the antigen (68). In theory, the B lymphocytes isolated from the peripheral blood of camelid animals can represent all the antibody genes in the animal, but as they have not been immunized with the antigen, the type and number of B lymphocytes in the body are unspecific, and the success rate of screening and the affinity of the obtained Nbs are lower.

Huo et al. reported that using the naïve (camel) library and PCR-based affinity maturation, two SARS-CoV-2 neutralizing Nbs (H11-D4 and H11-H4) were obtained and produced (69) (**Table 1**). H11-D4 and H11-H4 bind RBD with affinities of 39 and 12 nM, respectively, and prevent the interaction of RBD with ACE2. These two Nbs recognize the same epitope on RBD and partially overlap with the ACE2 binding surface, explaining that these two Nbs prevent the interaction of RBD and ACE2 (69) They and their Fc fusions show neutralizing activity against SARS-CoV-2 (H11-H4 is 4–6 nM, H11-D4 is 18 nM), and can cooperate with CR3022 to neutralize SARS-CoV-1/2 antibody (69).

The sources of Nbs obtained from immune or naïve libraries are camelid (or shark) animals. There are still rooms for improvement in the biophysical properties of antibodies (70). In particular, therapeutic antibodies have strict requirements, including thermodynamic stability, solubility, affinity, selectivity, oxidation, *etc.* All these properties must be optimized to achieve a balance of different biophysical properties, and its requirements far exceed those of natural antibodies in the organism.

Synthetic libraries use gene synthesis technology to introduce random DNA sequences at specific sites and become an alternative to naïve libraries. At present, some high quality large synthetic Nb libraries have been reported (71, 72), and some have been used to successfully screen high-affinity SARS-CoV-2 neutralizing Nbs (59, 73–75) (**Table 1**).

Walter et al. used three large-scale synthetic libraries (concave, ring and convex) to complete the rapid screening of SARS-CoV-2 Nbs within 12 working days through phage display and ribosome display, and obtained many highly effective SARS-CoV-2 neutralizing Nbs (75).

Researchers from Fudan University and Bio-Missile Corporation (China), based on the human heavy chain variable region (IGHV) sequence, humanized the Nb framework region and developed a humanized Nb phage library (74). They conducted a bio-panning for the SARS-CoV-2 RBD and S1 subunits and found that the two Nbs n3088 and n3130 can target the “hidden” epitopes located in the spike trimer interface, thereby effectively neutralizing SARS-CoV-2 (74).

Schoof et al. have developed a Nanobody (Nb6) that can inhibit the interaction of Spike and ACE2 through a synthetic library displayed on the surface of yeast (59). Nanobody Nb6 binds to Spike, locking its RBD domain in the down conformation which cannot bind to ACE2 (59). The trivalent mNb6-tri was produced through affinity maturation, structure-guided design and prokaryotic expression. The IC50 of mNb6-tri neutralizing new coronavirus is lower than pmol, and it can still maintain function after atomization, freeze-drying and heat treatment (59).

## Discussion

These studies have raised obvious expectations and hope that Nanobodies (Nbs) can be used in the treatment of COVID-19. Small size (almost a quarter of the human antibodies), simple structure, easy to use and relatively low cost, low immunogenicity and high affinity, making them special in research fields such as treatment, diagnosis and rapid diagnosis. Nbs seem to be very effective in capturing and stabilizing specific conformations, helping to gain a deeper understanding of biomolecular mechanisms and interactions. The highly stable Nbs can be nebulized and developed for use in inhalable preventive formulations, thereby ensuring that they are delivered directly to the lungs in the combat area. Another advantage is that even after long-term storage, their stability may not be sacrificed, so that Nbs can be reasonably stored and used as a treatment option in the event of pandemic such as COVID-19. Besides, multivalent Nbs or noncompeting Nbs cocktails may prevent mutations virus strains from escaping. Pymm et al. found that a Nb cocktail composed of two non-competitive Nbs can inhibit the interaction between ACE2 and RBD, and can effectively neutralize wild-type SARS-CoV-2 and N501Y D614G variants at relatively low concentrations (76).

In addition, the current rapid development of computational technology and artificial intelligence (AI) has promoted the development of protein structure prediction and computer-aided drug design. At present, large-scale co-evolution analysis is the commonly used algorithms for predicting the 3D structure of proteins based on gene sequences and performs quite well (77). It was used by Google’s “AlphaFold”, which can accurately predict protein structure based on protein sequence within a few days (78, 79).

A large number of high-quality antigen-antibody complex structures have been determined, coupled with the development of molecular simulation technology, the discovery of new antibodies by computers has become an important emerging field (70). With the development of theoretical chemistry and computational biophysics, we have a deeper understanding of the physical nature of protein folding and interaction, and various software for modeling and simulating protein and other biomolecules has been developed. Baker et al. used their Rosetta software to design a variety of proteins with unnatural structures (80) or proteins with high affinity to specific targets (81). The Rosetta software has also been extended to perform high-precision modeling of antibodies (82). Computer-aided Nb development may become a very important tool in the future. All in all, we believe that ongoing research will pave the way to a safer world.

## Author Contributions

FC perceived the conception, analyzed the findings, and wrote the manuscript. ZL and FJ assisted in writing the manuscript. All authors contributed to the article and approved the submitted version.

## Funding

This work was supported by the Guangdong Basic and Applied Basic Research Fund (Guangdong Natural Science Fund, grant no. 2019A1515110766, Grant from Guangdong Medical Science and Technology Research Fund (A2020280).

## Conflict of Interest

Author FJ was employed by the company NanoAI Biotech Co., Ltd.

The remaining authors declare that the research was conducted in the absence of any commercial or financial relationships that could be construed as a potential conflict of interest.
